# Canaloplasty using iTrack 250 Microcatheter with Suture Tensioning on Schlemm's Canal

**DOI:** 10.4103/0974-9233.56224

**Published:** 2009

**Authors:** Mahmoud A. Khaimi

**Affiliations:** Dean McGee Eye Institute, University of Oklahoma, Department of Ophthalmology, 608 Stanton L. Young Blvd, Oklahoma City, OK 73104

**Keywords:** Canaloplasty, iTrack microcatheter, Schlemm's canal

## Abstract

Open angle glaucoma (OAG) necessitating surgery has traditionally been treated with filtering procedures using antifibrotics. Unfortunately, such filtering procedures are not without the risk of postsurgical complications. Increasing interest in blebless surgery has led to innovative surgical procedures aimed at rejuvenating the natural trabeculo canalicular outflow pathway. Circumferential catheterization with suture tensioning of Schlemm's canal has emerged as a safe and effective way to surgically treat OAG.

## INTRODUCTION

Open angle glaucoma (OAG) is a disease which results in optic nerve damage with subsequent visual field loss due to ineffective drainage of aqueous humor through the eye's natural aqueous outflow channels. In the United States, OAG is typically treated first with topical pressure lowering drops followed by laser trabeculoplasty (LTP) and finally surgical intervention. The gold standard for the surgical treatment of glaucoma has historically been trabeculectomy with the use of antifibrotics. The trabeculectomy procedure serves to create a bypass route for the aqueous humor to drain out the eye and into a subconjunctival bleb. However, despite effectively lowering the intraocular pressure (IOP), trabeculectomy procedure is not without the risk of postsurgical complications.^1^–^6^ Postsurgical hypotony and a lifetime risk of blebitis are two of the most serious postoperative complications after a trabeculectomy procedure.^7^–^11^

**Figure 1:**
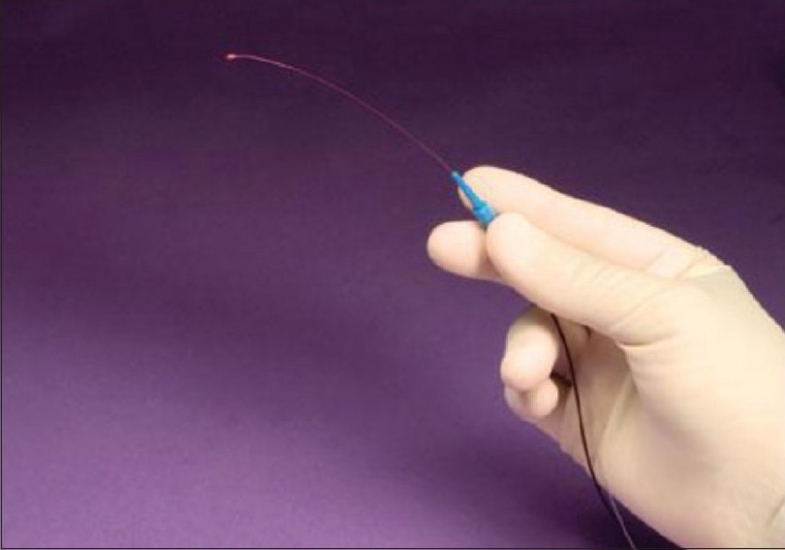
iTrack 250 Flexible Microcatheter (Courtesy, iScience Interventional, Menlo Park, CA)

More recently, increasing interest in rejuvenating the natural trabeculocanalicular outflow pathway has led to advancement in the surgical approach to treating OAG that avoids shunting aqueous to a nonphysiological drainage site and is less likely to result in postoperative hypotony. The iTrack 250 flexible microcatheter (iScience Interventional, Menlo Park, CA) has enabled the glaucoma surgeon to perform 360 degrees of canaloplasty under the direct visualization of a beacon lighted tip [Figure 1]. Unlike prior canaloplasty procedures, the iTrack device allows for circumferential viscodilation of the entire length of Schlemm's canal with subsequent suture placement through the canal. The suture allows for tension to be transmitted to the inner wall of Schlemm's canal and trabecular meshwork thereby restoring natural aqueous outflow.

## SURGICAL PROCEDURE

**Figure 2:**
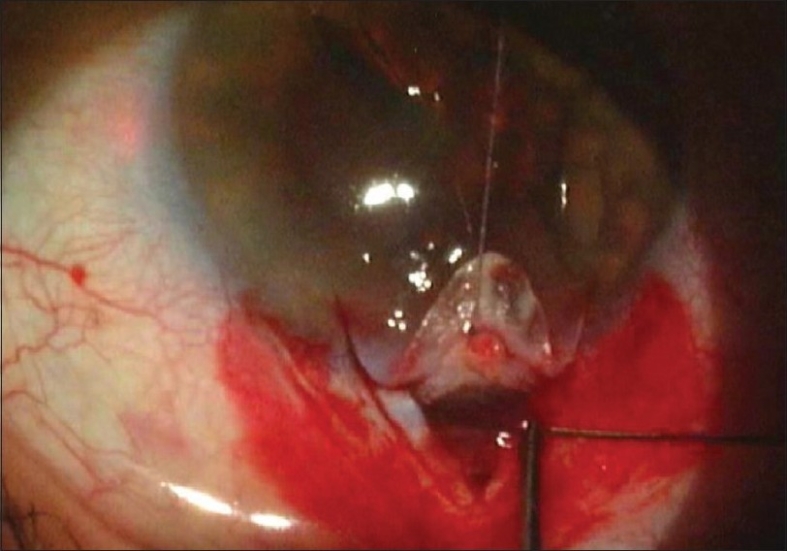
iTrack Microcatheter with Illuminating Tip in Schlemm's Canal (Courtesy, iScience Interventional, Menlo Park, CA)

The iTrack 250 flexible microcatheter is unique in two ways. One, the microcatheter has a beacon tip to allow for trans scleral illumination during catheterization of Schlemm's canal [Figure 2]. Secondly, the microcatheter has a 200 micron diameter shaft which is connected to an ophthalmic viscosurgical device (OVD) injector which permits precise injection of OVD while cannulating the canal.

**Figure 3:**
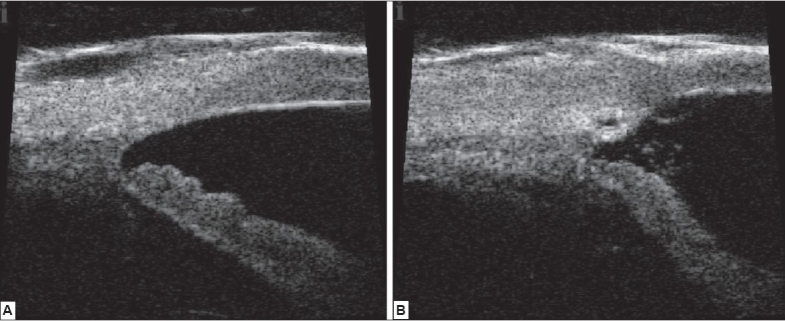
Images Captured with iUltrasound System: Same Eye before (A) and after (B) Canaloplasty. Increased Diameter of Schlemm's Canal postoperatively.

Typically, a fornix based conjunctival incision is created to allow for a superficial scleral flap followed by a deeper inner scleral flap to attain access to Schlemm's canal. The first flap is approximately 50% thick and the subsequent deeper flap is fashioned to expose and unroof Schlemm's canal. The canal's ostia are then viscodilated to allow for insertion of the microcatheter. The lighted tip allows for the surgeon to visualize the cannulation of Schlemm's canal for 360 degrees while the OVD injector allows for simultaneous injection of viscoelastic every two clock hours as the catheter is advanced. After complete circumferential catheterization of the canal the distal tip of the catheter emerges at the scleral cut down at which point a 10-0 polypropelene suture is tied to the tip. The microcatheter is then retracted pulling the suture in the canal. The suture is then cut away from the microcatheter and seated against the inner wall of Schlemm's canal and tied in a loop. Tension is then placed on the suture to maintain an inward radial force on the trabecular meshwork. High-resolution ultrasound biomicroscopy using the iUltrasound (iScience Interventional, Menlo Park, CA) is then utilized to visualize the amount of distention placed on trabecular meshwork in order to assess suture tensioning [Figure 3]. Once adequate tension is obtained, the suture is secured with locking knots. The descemetic window which was partially created while fashioning the deep scleral flap is now enlarged anteriorly. The deep scleral flap is then excised and the superficial flap is sutured watertight to avert bleb formation. The conjunctiva is then re approximated to limbus.

## DISCUSSION

Canaloplasty using the iTrack 250 microcatheter with suture tensioning of Schlemm's canal is a minimally invasive way to surgically treat OAG. Further, this procedure results in lower intraocular pressure (IOP) without the formation of a conjunctival bleb. As canaloplasty using the iTrack microcatheter with suture tension is a rather novel surgical approach to treating OAG very little data is available on the efficacy of such a procedure in comparison to the gold standard trabeculectomy. The largest study to date was recently published by Lewis *et al*. in a multicenter international prospective study to evaluate two-year postsurgical safety and efficacy of canaloplasty to treat OAG.^12^ The study included 127 eyes with OAG which were treated with canaloplasty suture tension alone or canaloplasty combined with cataract surgery. Eyes with canaloplasty alone had a mean IOP of 16.3± 3.7 mmHg and 0.6 ± 0.8 medications. Eyes with combined glaucoma–cataract surgery had a mean IOP of 13.4 ± 4.0 mmHg and 0.2 ± 0.4 medications. The investigators reported no serious postsurgical complications with 0.8% incidence of hypotony, 7.9% incidence of hyphema, 7.9% incidence of transient IOP spike of 30mmHg or greater during the immediate postoperative period, and microhyphema was observed in 8.7% one-day postoperatively.

Shingeleton *et al*. also analyzed the safety and efficacy of canaloplasty combined with clear corneal phacoemulsification with posterior chamber intraocular lens implant.^13^ As part of an international multicenter, prospective study 54 eyes that underwent combined canaloplasty and cataract surgery were evaluated. The mean baseline IOP was 24.4 mmHg ± 6.1 (SD) with a mean of 1.5 ± 1.0 medications per eye. In all eyes, the mean postoperative IOP was 13.6 ± 3.8 mm Hg at one month, 14.2 ± 3.6 mm Hg at three months, 13.0 ± 2.9 mm Hg at six months, and 13.7 ± 4.4 mm Hg at 12 months. Medication use dropped to a mean of 0.2 ± 0.4 per patient at 12 months. Surgical complications were reported in five eyes of which three had hyphema and one each had a Descemet tear and iris prolapse.

Canaloplasty using the iTrack microcatheter with suture tensioning of Schlemm's canal appears to offer the glaucoma specialist a surgically effective way to treat OAG by restoring the natural outflow pathway of the eye without the formation of a bleb or the complications associated with trabeculectomy. However, canaloplasty is not without limitations. The surgery is technically challenging and there is definitely a learning curve.^12^ In addition, not all patients with glaucoma are surgical candidates for canaloplasty. The procedure is contraindicated in eyes with angle recession, neovascular glaucoma, chronic angle closure, narrow angle glaucoma, and patients with previous ocular surgery that would prevent circumferential catheterization of Schlemm's canal.^14^ Although canaloplasty results in IOPs in the mid-teens, trabeculectomy has been shown to decrease IOP more significantly (16-18 in Godfrey). Further, clinical data shows that very low IOP is necessary to treat OAG.^15^–^17^ Canaloplasty outcome will also be limited in eyes in which the distal aqueous outflow channels are collapsed or scarred down. Therefore, canaloplasty seems to be a viable surgical option in patients with early OAG or ocular hypertension and traditional filtering surgery for more severe stages of the disease.
